# State and Trait Rumination Effects on Overt Attention to Reminders of Errors in a Challenging General Knowledge Retrieval Task

**DOI:** 10.3389/fpsyg.2020.02094

**Published:** 2020-09-02

**Authors:** Ronald C. Whiteman, Jennifer A. Mangels

**Affiliations:** ^1^Department of Psychology, Baruch College, The City University of New York, New York, NY, United States; ^2^Department of Psychology, Baruch College and The Graduate Center, The City University of New York, New York, NY, United States

**Keywords:** eye-tracking, gaze, brooding, reflection, feedback, fixation, RRS

## Abstract

Rumination is a recurrent and repetitive manner of thinking that can be triggered by blockage of personally relevant goals, creating a temporary state of abstract and evaluative self-focus. Particularly when focused on passive “brooding” over one’s problems and feelings, however, rumination can increase negative affect, interfere with problem-solving, and, through a negative feedback cycle, become a chronic trait-like style of responding to personal challenges, particularly in women. Given the pervasiveness of rumination and its potential impact on cognitive processes and emotional states, the present study asks how it impacts attention to feedback that either reminds individuals of goal-state discrepancies (reminders of errors) or could help to remediate them (corrective information). Using eye-tracking, we examined both state and trait rumination effects on overt measures of attention [first fixation duration (FFD) and total fixation duration (TFD)] during simultaneous presentation of these two types of feedback following failed attempts to answer challenging verbal general knowledge questions (average accuracy ∼30%). After a pre-induction baseline, we induced either a state of rumination using a series of writing exercises centered on the description of an unresolved academic concern or a state of distraction by centering writing on the description of a neutral school day. Within our women-only sample, the Rumination condition, which writing analysis showed was dominated by moody brooding, resulted in some evidence for increased initial dwell time (FFD) on reminders of incorrect answers, while the Distraction condition, which did not elicit any rumination during writing, resulted in increased FFD on the correct answer. Trait brooding augmented the expression of the more negative, moody brooding content in the writing samples of both Induction conditions, but only influenced TFD measures of gaze duration and only during the pre-induction baseline, suggesting that once the inductions activated rumination or distraction states, these suppressed the trait effects in this sample. These results provide some support for attentional-bias models of rumination (attentional scope model, impaired disengagement hypothesis) and have implications for how even temporary states of rumination or distraction might impact processing of academic feedback under conditions of challenge and failure.

## Introduction

Rumination is a recurrent and repetitive manner of thinking that can be triggered by blockage of personally relevant goals, creating a temporary state of abstract and evaluative self-focus that can eventually lead to a more chronic, trait-like style of ruminative responding to personal challenges (Watkins and Nolen-Hoeksema, 2014). Prominent models of rumination [Martin and Tesser’s (1996) Control Theory; Nolen-Hoeksema’s (1987) Response Styles Theory] describe both temporary (state) and chronic (trait) rumination as being maladaptive for both affect and goal-directed behavior (for a review, see Nolen-Hoeksema et al., 2008). This is particularly the case for the Ruminative Brooding (RB) subtype, which produces a sustained, but unproductive focus of attention on negative outcomes and their associated feelings. In contrast, the Reflective Pondering subtype is proposed to be more adaptive in nature because it taps the tendency to deliberately “reflect” on concrete means for problem solving (Treynor et al., 2003).

To date, few studies have examined the effects of either ruminative subtype within an academically relevant context (e.g., Lyubomirsky et al., 2003; Ciarocco et al., 2010). This is surprising given that students may face various types of cognitive and affective challenges during difficult performance assessments. For example, rumination is purported to underlie the detrimental social-cognitive phenomenon of stereotype threat (Beilock et al., 2007). The present study aims to address this gap in knowledge, specifically by examining how inducing a state of brooding-focused rumination in students influences selective attention to negative feedback during a challenging general-knowledge retrieval task (e.g., “What is the capital of Canada?”; Butterfield and Mangels, 2003; Whiteman and Mangels, 2016; Mangels et al., 2018). Here, we enlisted eye-tracking gaze metrics to examine how reminding individuals of past unattained academic goals through a narrative expression exercise (rumination induction) influenced selective attention to reminders of retrieval errors during the task. Specifically, we used fixation dwell time to evaluate selective attention when the correct answer was presented simultaneously and thus in *competition* with reminders of one’s recent mistake.

In their habit-goal framework of rumination, Watkins and Nolen-Hoeksema (2014) argue that it is not only internal events (e.g., negative affect experienced during bouts of dysphoria or depression) that can trigger rumination but also external events that are construed as impediments to goal attainment (e.g., negative environments, locations, and/or behaviors of others). Similarly, social-cognitive theories of rumination argue that bringing one’s immediate and personal goal-state discrepancies into awareness can cause momentary ruminative thoughts to come online and attention to be deployed toward related, self-relevant content, even among otherwise mentally healthy individuals (Martin and Tesser, 1996; Moberly and Watkins, 2008). Thus, if someone is already in a state of rumination, primed by reminders of past goal-state discrepancies, then receiving repeated negative feedback regarding task performance could be just the type of external event that might trigger maladaptive attentional patterns associated with greater internal and external attention to the error itself, rather than on remediating the error by focusing on corrective information (see also Nolen-Hoeksema and Morrow, 1991; Siegle et al., 2002; Moberly and Watkins, 2010).

### Using Narrative Expression to Induce Rumination

Roberts et al. (2013) found that instructions for research participants to dwell on an ongoing, real-world concern of theirs resulted in more ruminative thoughts about their concern during an unrelated go/no-go task than those who were instructed to focus on a resolved goal. As in Roberts et al. (2013), the current study asks students to initially identify their unresolved academic concerns but, unlike that study, then prompts them to descriptively write about (i.e., externally narrate) their concerns, thus adapting methodology used in expressive writing paradigms (e.g., Pennebaker, 1997; McAdams and McLean, 2013). The use of expressive writing provides a means for explicitly measuring whether participants are actively processing the state induction prompts and, furthermore, to quantify thoughts according to whether they are more brooding-like or more reflective in nature (Marin and Rotondo, 2017). In keeping with typical expressive writing methods, we pit expressive narration against a non-expressive condition in which individuals offer neutral narrations about how they spend a typical day in their schedule (e.g., Gortner et al., 2006; Sloan et al., 2008). Neutrally writing about a mundane daily routine creates an ideal comparison condition because it, similarly, focuses attention on an autobiographical episode in the academic domain, while at the same time keeping focus away from specific academic concerns.

Narrative expression can provide direct insight into participants’ thoughts and feelings, but not without the caveat that formulating and transmitting a coherent narrative to others may influence the framing of the situation itself. Past studies using expressive writing have yielded mixed outcomes for well-being and problem-solving in intervention-based studies (for a review, see Frattaroli, 2006). On the one hand, expressive writing can be a helpful, adaptive process and, by promoting self-affirmation through positive self-reflection (SR) and constructive meaning-making (e.g., Banks and Salmon, 2013; Cohen and Sherman, 2014), can offer writers an opportunity to confront, organize, and insightfully restructure their ongoing problems and issues (Lyubomirsky et al., 2006). Indeed, in some studies that use this kind of prompting, expressive writing has been shown to actually *reduce* rumination (Gortner et al., 2006; Sloan et al., 2008) and improve performance on exams (Ramirez and Beilock, 2011).

On the other hand, expressive writing can also be maladaptive, particularly when it is characterized more by unconstructive reasoning processes (Banks and Salmon, 2013), which instead focus writers’ attention on negative abstractions of the causes and undesirable consequences of their problems and prime more critical views of the self and fixed views of the situation (Lilgendahl et al., 2013). Not surprisingly, this latter style of expressive writing would seem to exemplify what it means to brood. Importantly, Marin and Rotondo (2017) have shown that the extent to which one’s expressive writing typifies brooding-like rumination rather than SR is linked with lower self-acceptance and more negative views of the self. Thus, it is conceivable that being prompted to write in such a way about an ongoing and unresolved academic (vs. being prompted to write about a typical day in one’s schedule) could bring about a brooding-like ruminative state that keeps attention negatively focused on the self and signals of one’s failures.

### Measuring the Effect of Rumination on Selective Attention to Negative Feedback

Past research has shown that trait Rumination is associated to a narrowed attentional focus onto negative, self-relevant information (Donaldson et al., 2007; Altamirano et al., 2010), as well as difficulty inhibiting and disengaging from negative information (Vanderhasselt et al., 2011), possibly because of repeated introspection on the perceived self-relevance of this material (Berman et al., 2010). With focus directed inwardly on negative self-referential thoughts, ruminators are less likely to retrieve potentially useful information during problem solving (Bernblum and Mor, 2010), or to process new, surrounding information (Levens et al., 2009). To explain these and other similar findings, various theories of attentional bias have been proposed, including the attentional scope model (Whitmer and Gotlib, 2013) and impaired disengagement hypothesis (Koster et al., 2011). In particular, the latter hypothesis suggests that negatively brooding over goal-state discrepancies impairs the ability to disengage attention from such troubles, at the expense of deploying attention elsewhere.

One way to explicitly measure how attention might be biased toward information that is perceived to be negative and/or self-relevant is through eye-tracking measures. Eye-tracking provides a direct and continuous measure of overt attention to visual stimuli by measuring exactly where individuals are looking (i.e., fixating their gaze) and for how long. Although this method can be particularly useful for informing questions regarding attention selection across multiple stimuli (see Armstrong and Olatunji, 2012), to date, only a few gaze fixation studies have been conducted for expressly studying the attentional mechanisms of rumination, and these have focused exclusively on trait rather than state forms of rumination (Duque et al., 2014; Owens and Gibb, 2017).

These findings implicate trait rumination in creating a maladaptive attentional bias toward negative stimuli, one that may serve to reinforce a ruminative, depressive mood. For example, Duque et al. (2014) found that higher trait rumination predicted a greater negative attentional bias in a free viewing study (i.e., more time spent processing sad and angry faces, but not neutral or happy faces), as measured by “total fixation duration” metric [i.e., TFD; the summed amount of time spent fixating an area of interest (AOI) while it is presented on-screen], even after controlling for depression. Similarly, Owens and Gibb (2017) found that greater *brooding-like* ruminative tendencies within a mentally healthy adult sample predicted increased dwell time on sad vs. happy faces. Interestingly, a recent eye-tracking study found that individuals who exhibited stable trait Brooding both at the time of study and over the course of the following year not only were slower to disengage attentional focus away from negative information but also were slower to engage attention with more positive stimuli (Allard and Yaroslavsky, 2019).

Given that habitual, trait-like forms of rumination may arise from repeated experience of more temporary states of rumination (Watkins and Nolen-Hoeksema, 2014), we might expect to observe similar patterns of attentional bias when individuals are temporarily induced to experience a ruminative state. To test this in the context of our academically relevant task, we presented trial-level feedback following attempts to answer general knowledge questions (e.g., What is the capital of Canada?) that consisted of an initial, centrally presented small circle colored to indicate response accuracy (red for incorrect, green for correct), followed by “competitive” answer feedback. Critically, if the participant’s answer was wrong, this competitive feedback would show the incorrect answer (e.g., Toronto) in red simultaneously with the correct answer (e.g., Ottawa) presented in gray, separated into the upper and lower halves of the screen such that they could not be fixated simultaneously (if the participant was correct, both halves would show the correct answer, with one in green, the other in gray).

During this competitive feedback, we predicted that following errors, participants induced into a state of rumination would demonstrate increased dwell time on reminders of their incorrect answer, compared to individuals induced to distract themselves away from academic concerns. Furthermore, attention to the reminder of the incorrect answer, which is informationally redundant with the initially presented accuracy feedback (i.e., red circle), could come at the cost of decreased dwell time to the simultaneously presented, but more informative correct answer. With regard to measuring dwell time, we used both TFD and first fixation duration (FFD). Although TFD is the more commonly used method in eye-tracking studies of rumination with faces (e.g., Owens and Gibb, 2017), given that the competitive answer feedback involved verbal stimuli, FFD may be better at isolating the participants’ initial lexical/semantic processing of the answers (Rayner and Duffy, 1986). In contrast, TFDs would inform the extent to which the participant fixed on the answers well after the meaning of those words had been acquired and potentially after exploring other parts of the display as well.

### Study Summary

In summary, the present study examined whether being induced to ruminate vs. distract impacts overt attention to competitive answer feedback in challenging general knowledge task, as measured by TFD and FFD. Using a novel induction task that was based on narrative expression, we hypothesized that participants who had been induced to think about an unresolved academic concern (i.e., Rumination condition) would be biased to dwell longer on potentially “rumination-congruent” reminders of the incorrect responses than individuals whose narratives had been directed to focus on a neutral, average day (i.e., Distraction condition). Additionally, greater attention to the incorrect answer might come at the cost of attention to the correct answer, despite the latter’s greater information value.

We note that even though our primary interest was in whether students without clinical depression might show evidence of negatively biased attention when reminded of unmet academic goals, many previous rumination induction studies find the most adverse effects for individuals who are concurrently in a depressive mood state (for a review, see Nolen-Hoeksema et al., 2008). Thus, we assessed trait rumination and depression in order to examine whether these individual difference factors interacted with our state-level manipulations of attention. Past studies have also suggested that gender may play a role in defining the effects of rumination, in that women more than men tend to ruminate over their affective state in the face of negative outcomes and difficult life events (Nolen-Hoeksema and Jackson, 2001; Mezulis et al., 2002; Johnson and Whisman, 2013). Although past studies of rumination induction have not reported effects of gender (e.g., Lyubomirsky and Nolen-Hoeksema, 1995; Lyubomirsky et al., 1998; Lyubomirsky et al., 2003), the particular general knowledge task used in this study often demonstrates stronger effects in women with regard to both manipulations of context and individual difference variables compared to men (see Whiteman and Mangels, 2016; Mangels et al., 2018; Abraham et al., 2019). Therefore, we felt that restricting our sample to women would provide the most robust test of our hypotheses, even if it would limit the generalizability of the results.

## Materials and Methods

### Participants

Fifty-nine women were recruited from the Baruch College undergraduate population via the institution’s research participation subject pool. They ranged in age from 18.0 to 34.2 years (*M* = 20.50, *SEM* = 0.40), self-reported being native English speakers or fluent by age 6, had normal or corrected-to-normal hearing and vision, and had no history of eye disorders (e.g., detached or torn retina, macular degeneration, glaucoma, color blindness). To limit our sample to students without clinically significant depression, while still including a representative range of participants, an additional inclusion criterion was that they scored 19 or lower on the Beck Depression Inventory II (BDI-II; Beck et al., 1996). As compensation for their participation, subjects received either research credit as part of a course requirement (69.5%), monetary compensation at a rate of $10/h (8.5%), or some combination of both credit and money (22.0%).

Four participants were excluded from both the behavioral and eye-tracking analyses (three Distraction conditions and one Rumination condition) because they did not have enough semantic error trials for analysis. In particular, two participants self-terminated before Block 2, one participant performed 1.5 times the upper interquartile range of scores in the first two blocks, and one participant was an outlier with regard to orthographic errors (26%, as confirmed by a boxplot outlier analysis)^1^. Additionally, four more subjects (two from each Induction condition) did not have a minimum number of usable eye-tracking trials (minimum = 3; Chua et al., 2012) in one or more conditions after pre-processing to remove trials with excessive signal loss or initial central fixation failure (see section “Eye Tracking” for details), necessitating their removal from the eye-tracking analyses, although they were retained for behavioral analysis. Exclusion of these subjects resulted in 55 subjects for behavioral analysis and 51 subjects for the eye-tracking analysis.

Table 1 shows the distribution of these participants across Induction conditions, as well as their group characteristics. For both analysis groups, there were no condition differences in BDI-II scores, or in trait levels of rumination as measured by either the overall Ruminative Responses Scale (RRS; Nolen-Hoeksema and Morrow, 1991) or the Brooding and Reflection subscales^2^ (all *p*s > 0.26). However, the two groups did differ marginally in their age [behavioral sample: *t*(53) = 1.93, *p* = 0.06; eye-tracking sample: *t*(49) = 1.78, *p* = 0.08] and in years of education [behavioral sample: *t*(53) = 2.38, *p* = 0.02; eye-tracking sample: *t*(49) = 1.83, *p* = 0.07]. However, both differences were small in actual magnitude, amounting to less than 1.5 years of age and one semester of education.

**TABLE 1:** Sample characteristics with mean scores of pre-test self-report questionnaires and demographics.

	**Behavioral sample**		**Eye-tracking sample**	
**Variable**	**Rumination**	**Distraction**	**Rumination**	**Distraction**
*n*	28	27	26	25
Age	21.33 (0.72)	19.77 (0.34)	21.36 (0.78)	19.81 (0.36)
Years of education	13.94 (0.22)	13.22 (0.20)	13.86 (0.23)	13.28 (0.21)
BDI-II	6.64 (0.90)	8.33 (1.18)	6.69 (0.95)	7.84 (1.20)
RRS Total	39.57 (2.00)	42.78 (2.06)	40.07 (2.12)	42.60 (2.23)
Brooding	9.71 (0.71)	10.67 (0.67)	9.88 (0.75)	10.52 (0.71)
Reflection	9.46 (0.69)	9.22 (0.59)	9.54 (0.74)	9.24 (0.63)

**Standard errors of the mean appear in parentheses in this and all other tables.**

### Materials

#### General Knowledge Stimuli

Stimuli consisted of 138 items from a larger, previously normed pool of 406 general knowledge question and answer stimuli^3^. These were divided into two bins of 69 items for use in each block of the general knowledge task. Bin order was counterbalanced across blocks within each Induction condition. Using information from the normed database, questions for each bin/block were matched for average difficulty (i.e., target accuracy of 0.35). The length for all correct answer words was pre-set to range from four to nine letters (Bin 1: *M* = 6.36, *SD* = 1.16; Bin 2: *M* = 6.44, *SD* = 1.31), which typifies the word length shown elsewhere to only require a single fixation for effective lexical processing (Rayner, 1979, 1984). We also ensured that all correct answer word stimuli had been rated previously as being familiar to 95% of the Baruch College undergraduate population, thus reducing the likelihood that large variations in semantic word fluency would influence gaze fixation behavior (Gernsbacher, 1984; Rayner and Duffy, 1986; Shatzman and McQueen, 2006).

#### Software and Hardware

The general knowledge task was delivered using Presentation software (Neurobehavioral Systems, Inc., Berkeley, CA) and programmed to sync up and interface with Tobii Studio Software (Version 2.3.1; Tobii Technology, Inc., Falls Church VA) in a dual-computer setup. In the testing booth, the general knowledge stimuli were presented on a 23″ 1080 screen resolution; 60 Hz refresh rate) that was part of a Tobii TX300 integrated eye tracker system, which recorded gaze data at a sampling rate of 300 Hz. Data were pre–processed using Tobii Studio software and then exported and processed further in Matlab (Mathworks, Natick, MA, United States) using an in–house script.

### Design and Procedure

#### Overview

**FIGURE 1:**
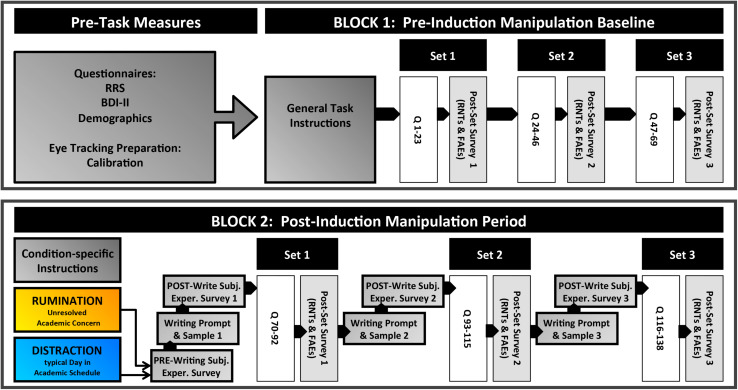
Overview of study procedures. Please refer to section “Overview” for detailed explanation.

The complete study procedure, from initial pre–task measures through the final task block, are illustrated in Figure 1.

Following informed consent, subjects first filled out the pre–task questionnaires, including the RRS, the BDI–II, and a set of demographic questions. They were then escorted to a well–lit room where they were seated comfortably, without a chin rest or head constraints, approximately 60 cm in front of the integrated Tobii computer monitor/eye tracker system. A nine–point calibration procedure was carried out to establish eye position.

Prior to Block 1, all subjects were presented with general task instructions detailing the basic structure of a general knowledge trial. Block 1, which served as the pre–induction manipulation baseline, was subdivided into three 23–question sets (for individual question trial structure, see section “Trial Sequence”). Each question set was followed by a short survey of the subjects’ subjective experiences during the preceding set (see section “Post–set Surveys”), including the extent to which they experienced recurrent negative thoughts (RNTs) and had negative feelings after errors (FAEs).

After completing Block 1, subjects began Block 2, which was defined as the post–induction manipulation period. This block began with *Condition–Specific Instructions* (see section “Condition–Specific Instructions”) regarding a writing–based task where participants were prompted to retrieve from autobiographical memory either an ongoing and unresolved negative academic situation from their life (Rumination condition) or a situation from a non–emotional, typical academic day (Distraction condition). After identifying an appropriate situation, they completed a *pre–writing* survey that queried the degree of concerned thinking they had recently experienced about their situation of focus. Then, they engaged in writing short narrative responses (3–5 min) to a specific prompt (see section “Induction–Related Writing Prompts”). This was followed by a rating of their *post–writing* subjective experiences about the writing task itself. They then engaged in answering a set of 23 general knowledge questions. As in Block 1, each question set was followed by the post–set survey of subjective experiences. This sequence, from the writing sample to the post–set surveys, was repeated for two more sets for a total of three sets (69 questions) in Block 2.

#### Trial Sequence

**FIGURE 2:**
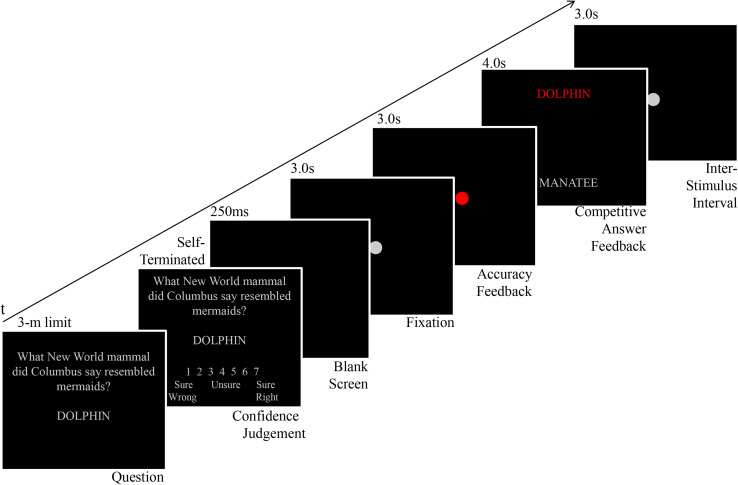
Trial structure. Example shows a trial with an incorrect answer. If the subject’s answer had been correct (i.e., Manatee), a green circle and green correct answer would have been shown, along with the correct answer in gray.

As shown in Figure 2, for each individual trial, questions were presented in gray font on a black background. Subjects had a 3–min time limit to submit their response, after which they rated their response confidence on a scale from 1 (sure wrong) to 4 (unsure) to 7 (sure right). They were then presented with a short blank screen for 250 ms, followed by a 3–s fixation period, consisting of a screen–centered gray circle that subtended 1° of visual angle (VA). Then, an initial indicator of participants’ performance accuracy was presented for 3 s, also consisting of another centered circle, 1° VA in diameter, where the color red indicated an error response and the color green indicated a correct response.

Immediately after, the competitive answer feedback was shown. The correct answer was always presented in gray, but participants’ responses were presented in green if correct and in red if incorrect. These two answers were presented in center–justified, vertical alignment, separated by approximately 19.1 cm and subtending at about 17° VA. Whether the gray, task–given correct answer appeared on the top of the screen or at bottom on any given trial was pseudorandomly counterbalanced for correct and incorrect trials separately, such that a given answer type did not consecutively appear in the same location for more than three trials.

Each word stimulus was 1.1 cm (∼1°VA) tall and could be as wide as 9.9 cm (i.e., nine letters long, or ∼9°VA), but as narrow as about 4.4 cm (i.e., four letters long, or 4°VA). This competitive answer feedback was presented for 4 s, a duration consistent with other eye–tracking studies using a competitive, free–viewing stimulus design (Kellough et al., 2008; Duque et al., 2014; Owens and Gibb, 2017). After offset of the competitive answer feedback, an inter–stimulus interval ensued, consisting of a 3–s presentation of a screen–centered gray circle, subtending 1°VA.

#### Post–set Surveys

After each 23–item set of general knowledge questions, we asked participants to rate the frequency of their RNTs and the relative pleasantness of their FAEs. Each post–set survey question was rated on a 1–9 Likert scale, with 1 reflecting the negative or low end of the subjective experience, 9 reflecting the positive or high end, and 5 indicating an experiential midpoint (i.e., neutrality).

#### Condition–Specific Instructions

Just prior to the onset of the induction manipulation in Block 2, the following general statement was presented to all participants:

“*Based on your responses so far, you encountered some difficulty answering the first block of questions. Although this difficulty is happening within the context of this research study, perhaps you have encountered difficulties in actual academic situations of your own life. During real–life academic difficulties, many students often report taking the time to*….”

This final phrase was completed by condition–specific instructions to either “*think about their academic difficulties in real life*” and identify an ongoing and unresolved academic concern of theirs that had come about recently and was currently causing them distress (Rumination Condition) or to “*distract themselves from the general knowledge questions or any real–life academic difficulties*” by identifying a recent non–emotional day in their academic schedule for which they could remember with good accuracy the events that took place (Distraction Condition). To help participants with this process, they were supplied with two condition–specific, scenario–based examples of suitable situations of focus, described as being previously offered by actual participants (see *Supplemental: Condition–Specific Instructions* for the full set of condition–specific instructions and *Supplemental: Condition–Specific Examples* for the condition–specific scenario–based examples).

Once participants identified their situation of focus for the writing task, they briefly described it to the experimenter who ensured it was suitable for their condition. If it was not suitable, or they struggled to identify one at all, participants were redirected with a few verbal prompts until they were successful. Participants were then asked to complete a short, condition–specific four–question *pre–writing* survey that queried the degree of concerned thinking they had recently experienced about their situation of focus on a 9–point Likert scale with 1 indicating low levels of concern and 9 indicating extreme levels of concern (see *Supplemental: Pre–Writing Survey* for the full set of items).

#### Induction–Related Writing Prompts

After a suitable situation had been identified, but prior to starting each of three 23–item sets of questions in Block 2, participants were presented with condition–specific prompts on how to craft their writing samples.

For the Rumination condition, the writing prompts were based on three important assumptions of Martin and Tesser’s (1996) Control Theory of rumination. First, given their claim that rumination is born out of goal–state discrepancies that are persistent and revolve around a common instrumental theme, the first prompt asked participants to factually describe with as little emotional expression as possible what their ongoing academic concern was and why it seemed to be persisting. Second, given that rumination over unresolved goals can be passive and automatic, the next prompt asked participants to describe the kinds of recurrent and repetitive thoughts that tended to easily come to mind about their ongoing academic concern. Third, given that rumination comes online when the rate of progress toward a goal is slower than what the individual wants it to be, the final prompt asked participants to describe the degree of investment of their time and energy that had been expended in vain in attempts to resolve their concern.

For the Distraction condition, the prompts were fashioned based on “fact control writing” often used in the expressive writing literature (e.g., Seeley et al., 2017), where participants write a factual account with little to no emotion of a recent day in their schedule. Thus, the first prompt asked participants to factually describe the events of a recent, non–emotional day from their academic schedule. The second prompt asked them to describe precisely *when* in the day (i.e., at what exact time) the events they had previously described occurred. Finally, the third prompt asked participants to describe precisely *where* they were (i.e., at what exact spot on or around campus) when the aforementioned events they had described occurred (see *Supplemental: Induction–Related Writing Prompts* for exact wording of prompts for both Rumination and Distraction conditions).

The prompts in each condition were always presented in the order mentioned above (i.e., no counterbalancing was used), given that this particular sequence was deemed ideal for creating a natural and continuous mental thread. To further facilitate continuity, participants’ previous writing samples were shown to them prior to completion of the next one. When completing all writing prompts, participants were also asked to constrain their focus to *past–oriented* thinking about their situation. This was particularly important for the Rumination condition because, although ruminating about past negative events is commonly associated with concern for the future (Watkins et al., 2015), such future–oriented, recurrent, and repetitive negative thinking is more often described as being a form of “worry” than rumination (Martin and Tesser, 1996; Watkins, 2008).

After writing for each prompt, participants were asked to fill out a short *post–writing* survey that queried their subjective experiences while completing that particular writing sample. The five items for the survey were selected from the “experiential self–focus” rumination induction prompts originally developed by Nolen–Hoeksema and Morrow (1993) and adapted for use in survey form. Using a scale that ranged from 1 to 9, with 1 reflecting an extreme amount of a given negative self–focus characteristic (hopelessness, restlessness, sadness, agitation, and fatigue), 9 reflecting an extreme amount of the corresponding positive self–focus characteristic (e.g., hopefulness, calmness, happiness, relaxation, and energy), and 5 indicating a middle, neutral point.

### Data Analysis

#### ANCOVAs

We conducted a series of customized analyses of covariance (ANCOVAs) that included the categorical factors of Induction condition, Set (except on measures of eye–tracking due to trial counts; see below), and Block (except on measures of the writing exercise, which only occurred in Block 2), alongside subjects’ continuous BDI–II scores and RRS Brooding and Reflection scores as covariates (i.e., predictor variables). Although parameter estimates were rendered for all three covariates, including all interaction terms between each of the covariates and manipulated variables, the ANCOVAs did not include interaction terms between Brooding, Reflection, and BDI–II themselves, given that these trait/mood covariates were included mainly to determine if state effects were present even when controlling for these effects and/or they moderated any observed state effects. Additionally, prior to entering eye–tracking and subjective experience metrics into these ANCOVAs, we partialed out any variance associated with differences in performance accuracy on the general knowledge task in order to control for differences in accuracy, given that basic differences in error frequency could influence our key eye–tracking metrics^4^. This was done to increase the likelihood that any observed differences in those variables were the result of the induction and not individual differences in general knowledge.

For all analyses, an alpha level of *p* < 0.05 was used as criterion for significance, but marginally significant findings (0.05 ≤ *p* < 0.10) regarding experimental manipulations are also reported and explored because of *a priori* predictions with these factors. On the other hand, marginal effects involving trait and mood effects are only reported given that these analyses were highly exploratory. Effect sizes are specified in all cases using the partial eta squared statistic. Where necessary, Greenhouse–Geisser corrections were used for violations of sphericity, and where appropriate, linear trend analyses were conducted for the three-level within-subjects factors of “Set” or “Prompt” to especially explore how differences may have unfolded within the post-induction period of the task. Any *post hoc* explorations of significant main effects or interactions were carried out using the Holm–Bonferroni procedure for corrections for multiple comparisons (Holm, 1979).

#### Eye-Tracking

**FIGURE 3:**
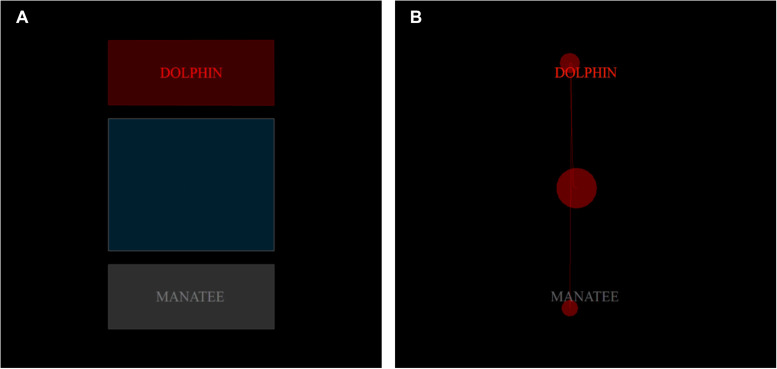
Competitive answer feedback AOIs and sample eye–tracking data. **(A)** Areas of interest (AOIs) are superimposed over each feedback type (red = incorrect subject–given answer, gray = correct task–given answer, blue = initial central fixation area). **(B)** A short snippet (<1 s) of eye–tracking data from a single subject is superimposed to illustrate fixations (red circles, where diameter is a function of duration) and saccades (thin red lines). During the task, the participants were free to view the competitive answer feedback for a 4 s period.

For each trial, a static, rectangular-shaped AOI with a width of 14.1 cm (12.5°VA) and a height of 5.6 cm (5°VA) was centered over each of the two pieces of word feedback for the full duration of their presence on-screen (i.e., 4 s, see Figure 3 for a pictorial representation of the AOIs used in the current study). Since any one word stimulus itself was 1.1 cm (∼1°VA) tall, and could be as wide as 9.9 cm (i.e., nine letters long, or ∼9°VA), the overlay of the AOI centrally on top of the longest possible word stimuli (including participants’ typed responses) permitted a buffer of additional screen space of 2.1 cm (i.e., ∼2°VA) from the outer borders of nine-letter word stimuli out to the edge of the AOI in any direction. These AOIs were used for the word stimuli for every trial, regardless of word length.

AOIs with these kinds of parameters have been used elsewhere in other studies investigating visual fixations of word stimuli (Dampuré et al., 2014). Although seemingly conservative, these AOI parameters were also chosen given that during normal reading, the information necessary for making accurate semantic assessments of fixated word stimuli is limited to foveal vision (Rayner, 1979), and central foveal vision can subtend up to 5°VA (Duchowski, 2007). Given that Hansen and Ji (2010) report the error rate for the accuracy of model-based gaze estimation systems like that of the Tobii TX300 to be between 1° and 2°VA, applying such conservative parameters for the two word feedback AOIs in our study likely helped account for any degree of this technical uncertainty.

Whether participants looked at the two pieces of word feedback on any given trial (and for how long) was defined by assessing the degree of gaze fixation (expressed in ms accrued) that occurred within each of the two AOIs. In accord with standard settings used on current Tobii eye trackers, fixations were defined using the Velocity-Threshold Identification (I-VT) fixation classification algorithm, where a velocity threshold of any directional shift of the eye that was below 30 visual degrees per second across data points was used to operationalize a single fixation. To preserve the continuity of gaze data in momentary instances (i.e., <75 ms) of signal loss, a gap fill-in interpolation algorithm was applied, and any adjacent fixations found to be within 0.5° VA of one another were merged. Any defined fixation ultimately determined to be shorter than 60 ms in length was re-classified as saccade data.

Any trial was excluded from analysis if gaze fixation was not centered between the two feedback AOIs at the start of the trial in a region 11.75 cm (10.5° VA) in height and 14.1 cm (12.5° VA) in width (see Figure 3; left panel). We also excluded any trial from analysis where the summation of available fixation time for that trial was less than 2.67 ms (i.e., two thirds the duration of competitive feedback presentation), or if that time was more than 2 SD below the participant’s mean summed-fixation time across all trials (e.g., Chua et al., 2012). After these exclusions, if we had retained set as a factor, there would have been 17 participants excluded, and therefore we opted to collapse over this factor to retain the maximal number of participants. Even after collapsing, four subjects did not have a minimum of trials in all critical conditions (as a function of answer type, answer location, and task block), necessitating their exclusion from all further analyses.

Our measures of interest were FFD, defined as the time spent looking at an answer AOI the first time it was fixated upon, and TFD, defined as the overall time in ms, summed across all fixations, spent looking at an answer AOI. FFD and TFD values were generated for each of the two AOIs in every single trial, after which single-subject averages of each gaze fixation metric were calculated for error trials as a function of answer type [subject-given incorrect answer (red) vs. task-given correct answer (gray)], task block (Block 1 or 2), and Induction condition (Rumination or Distraction).

Only those error trials that represented semantic errors were included for analysis, thereby excluding orthographic errors (see Footnote 1) and correct trials. There were two reasons why we did not include correct answers in our analyses. First, in order to make the answer feedback for correct and incorrect trials visually similar, feedback on a correct trial had to include the correct answer in both positions on the screen, with one in green and one in gray. As a result, there was no meaningful competition for attention between the two answers in terms of information, only in terms of color. Thus, any looking-time differences to one of these correct answers would be based purely on color and would not inform our primary research questions. Second, the experiment was purposefully designed to have more incorrect than correct trials so that participants would experience challenge and difficulty throughout the task. This gave us enough incorrect trials to withstand some trial loss due to signal drop out, but too few correct trials for analysis.

We also initially calculated FFD and TFD as a function of Answer location (i.e., whether the gray, task-given correct answer was presented at the top of the screen or at the bottom). However, prior to conducting the main eye-tracking analyses of interest, we determined that we could simplify our statistical model by collapsing across this factor because the number of useable trials at each location did not interact with condition and/or block (all *p*s > 0.26), and thus, any effect of location should influence the Rumination and Distraction conditions equally. Although there were no significant differences in the number of trials across conditions (*p* > 0.91), there were significantly more trials in both conditions that were retained in Block 1 (Rumination: *M* = 31.5, *SEM* = 2.02; Distraction: *M* = 32.5, *SEM* = 2.06) compared to Block 2 (Rumination: *M* = 29.7, *SEM* = 1.89; Distraction: *M* = 28.1, *SEM* = 1.93), *F*(1, 49) = 7.43, *p* = 0.009, η*_p_* = 0.132.

## Results

### Rumination and Distraction State Inductions

#### Pre-writing Survey

**FIGURE 4:**
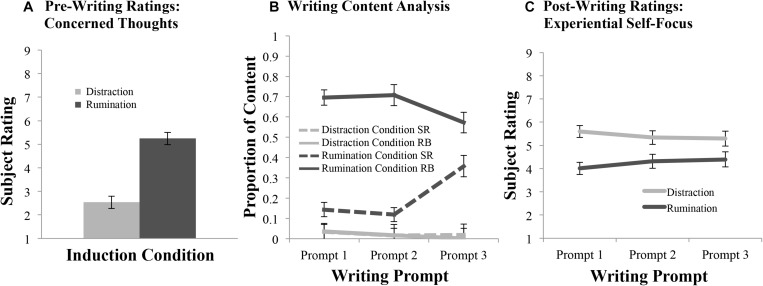
Writing task ratings. **(A)** Subjects’ average pre-writing ratings of the degree of concerned thinking (1 = low, 9 = high) regarding the situation of focus for the writing-based task. Ratings are plotted as a function of Induction condition (Distraction in light gray; Rumination in dark gray). **(B)** The proportion of ruminative writing content (*Y*-axis), whether in Ruminative Brooding (RB; solid lines) or Self-Reflection (SR; dotted lines) type, is plotted as a function of Induction condition (Distraction in light gray; Rumination in dark gray) across each of the three Writing prompts during the post-induction period. **(C)** Subjects’ average post-writing ratings of the valence of experiential self-focus (1 = extreme negative amount, 9 = extreme positive amount) during the writing-based task, as a function of Induction condition and Writing prompt.

Following identification of their condition-specific situation, but before beginning their first writing sample, participants self-reported their degree of concern regarding the situation they would be writing about. In line with expectations, identifying an unresolved negative academic situation (Rumination condition) generated more concern than identifying a non-emotional day in one’s academic schedule (Distraction condition), *F*(1, 47) = 53.72, *p* < 0.001, η*_p_*^2^ = 0.53 (see Figure 4A). Neither BDI-II nor RRS subscores were associated with the strength of these ratings, whether overall or via interaction with the Induction condition factor (all *p*s > 0.38).

#### Writing Content Analysis

Participants’ three induction-related writing samples were rated sentence by sentence for RB and SR writing content types according to the coding system of Marin and Rotondo (2017). Briefly, RB was any negative statement that described an undesirable outcome/consequence, its cause, or any negative evaluation. SR was any positive or neutral statement that provided an evaluation/explanation about the self, others, or the self–other relationship. Also included were statements that provided constructive or insightful reasoning toward problem-solving or any adaptive action toward resolving one’s concerns. Because one sentence could contain more than one phrase, each one capturing a *different idea*, it was possible that one sentence could be coded as containing both RB and SR content. However, in cases where it was deemed that the participant wrote both RB and SR content about the *same idea* in the same sentence, that sentence was coded as only containing either RB or SR content, based on whichever type was expressed as the concluding remark. Upon the completion of coding, within any single writing sample, the number of sentences containing RB and SR content were each then separately summed and divided by the total number of sentences written, thus rendering separate, but non-mutually exclusive proportions for each rumination content type in that writing sample. Additional details regarding this coding can be found in *Supplemental: Writing Content Analysis*.

An ANCOVA that included the categorical factors of Induction condition, Rumination content type (RB vs. SR), and Writing prompt (three prompts) alongside the continuous trait covariates (see section “Data Analysis” above) demonstrated a significant three-way interaction involving all categorical factors, *F*(2, 94) = 3.63, *p* = 0.032, η*_p_*^2^ = 0.07, which subsumed several significant two-way interactions [Induction condition by Rumination content type: *F*(2, 94) = 29.09, *p* < 0.001, η*_p_*^2^ = 0.38; Induction condition by Writing prompt: *F*(2, 94) = 3.18, *p* = 0.046, η*_p_*^2^ = 0.06; Rumination content type by Writing prompt: *F*(2, 94) = 4.53, *p* = 0.013, η*_p_*^2^ = 0.09], as well as main effects of Induction condition, *F*(1, 47) = 714.32, *p* < 0.001, η*_p_*^2^ = 0.94, and Rumination content type, *F*(1, 47) = 27.91, *p* < 0.001, η*_p_*^2^ = 0.37. Below, we unpack the three-way interaction, whose results largely validate the effectiveness of the induction manipulation in expected ways.

First, as shown in Figure 4B, RB content was significantly greater for the Rumination condition compared to the Distraction condition following all three writing prompts (Prompt 1: *p* < 0.001; Prompt 2: *p* < 0.001; Prompt 3: *p* < 0.001). On the other hand, although SR content was low and did not differ between Rumination and Distraction conditions following the first two prompts (*p*s > 0.53), by the third prompt, participants in the Rumination condition included significantly more SR in their writing than those in the Distraction condition (*p* = 0.001). Indeed, following the third prompt, RB and SR rates were not statistically different within the Rumination condition after correcting for multiple comparisons (*p* = 0.54). This was *not* due to a substantial decrease in RB content here compared to the earlier two writing samples (all *p*s > 0.42 after correction), but rather to an increase in SR content (Prompt 1 vs. 3: *p* = 0.003; Prompt 2 vs. 3: *p* < 0.001).

Next, we evaluated the role that trait rumination and mood state may have played in the expression of ruminative content in the writing samples. The only significant finding associated with trait Brooding was a main effect of *increased* ruminative expression overall, regardless of Induction condition, Content type, or Writing prompt, *F*(1, 47) = 9.65, *p* = 0.003, η*_p_*^2^ = 0.17. However, for trait Reflection, a significant three-way interaction emerged that included Induction condition and Writing prompt, *F*(2, 94) = 4.27, *p* = 0.017, η*_p_*^2^ = 0.08. Specifically, trait Reflection was associated with a *significantly reduced* amount of ruminative expression (collapsed across ruminative Content type), but only in the Rumination condition, and only after the third prompt, β = −0.58, *t* = 3.88, *p* < 0.001, η*_p_*^2^ = 0.24. Although we found a marginally significant three-way interaction involving pre-task mood (i.e., BDI-II scores), Induction condition, and Writing prompt, *F*(2, 94) = 2.55, *p* = 0.083, η*_p_*^2^ = 0.05, that also subsumed a marginally significant two-way interaction involving BDI-II and Writing prompt, *F*(2, 94) = 2.55, *p* = 0.083, η*_p_*^2^ = 0.05, these will not be explored further (see section “ANCOVAs”).

#### Post-writing Ratings

An ANCOVA demonstrated a significant two-way interaction effect between Induction condition and Writing prompt on post-writing experiential self-focus, *F*(1.69, 79.32) = 7.79, ε = 0.84, *p* < 0.001, η*_p_*^2^ = 0.14, which subsumed a significant main effect of Induction condition, *F*(1, 47) = 8.47, *p* = 0.006, η*_p_*^2^ = 0.15. *Post hoc* tests of the two-way interaction revealed that only after the first writing exercise did the Rumination group evidence significantly more negative self-focus than the Distraction group (see Figure 4C). However, there was an overall trend for self-focus to move toward neutrality in both groups, as was confirmed by exploration of a significant two-way linear trend effect involving Induction condition and Writing prompt, *F*(1, 47) = 9.87, *p* = 0.003, η*_p_*^2^ = 0.17. Whereas those induced to ruminate reported experiencing self-focus that was initially “somewhat negative” but then trended upward toward being more neutral, those induced to distract initially reported self-focus that was “somewhat positive” but then trended downward toward neutrality. Thus, although those induced to ruminate experienced more negative self-focus than those induced to distract, this difference was only prominent earlier in the post-induction period.

Turning to the possible influence of pre-task levels of RRS and depression, we found a marginal three-way interaction involving Induction condition, Writing prompt, and Brooding, *F*(1.69, 79.32) = 3.30, ε = 0.84, *p* = 0.073, η*_p_*^2^ = 0.07, and a significant two-way interaction effect involving trait Reflection and Writing prompt, *F*(1.57, 79.32) = 5.71, ε = 0.84, *p* = 0.019, η*_p_*^2^ = 0.11. Exploration of this latter significant interaction yielded no significant parameter estimates (all *p*s > 0.14). No effects involving pre-task BDI-II levels were found (all *p*s > 0.55).

### General Knowledge Task Performance

#### Task Accuracy

When we submitted task accuracy rates to our ANCOVA, no main effects or interactions of Induction condition, Block, or Set emerged (all *p*s > 0.18; see Table 2). Although we did find a significant three-way interaction involving Induction condition, Block, and trait Brooding, *F*(1, 47) = 4.99, *p* = 0.030, η*_p_*^2^ = 0.10, as well as Induction condition, Block, and BDI-II, *F*(1, 47) = 4.12, *p* = 0.048, η*_p_*^2^ = 0.08, none of the *post hoc* comparisons associated with these interactions survived Holm–Bonferroni corrections (all *p*s > 0.24).

**TABLE 2:** Task accuracy and ratings of task-related recurring negative thoughts (RNTs) and feelings after errors (FAEs), as a function of Induction condition and Block and Set.

		**Block 1 (pre-induction baseline)**			**Block 2 (post-induction period)**		
**Variable**	**Condition**	**Set 1**	**Set 2**	**Set 3**	**Set 1**	**Set 2**	**Set 3**
**Accuracy**							
	Rumination	0.323 (0.022)	0.280 (0.028)	0.280 (0.024)	0.290 (0.023)	0.300 (0.021)	0.300 (0.024)
	Distraction	0.325 (0.023)	0.347 (0.029)	0.307 (0.024)	0.318 (0.023)	0.345 (0.022)	0.310 (0.024)
**RNTs**							
	Rumination	3.80 (0.36)	3.93 (0.36)	4.19 (0.37)	2.90 (0.35)	2.81 (0.38)	2.76 (0.37)
	Distraction	4.05 (0.37)	4.05 (0.36)	3.56 (0.38)	3.63 (0.36)	3.37 (0.38)	3.23 (0.37)
**FAEs**							
	Rumination	4.09 (0.24)	3.62 (0.26)	3.44 (0.28)	4.20 (0.23)	3.91 (0.27)	3.92 (0.23)
	Distraction	3.76 (0.25)	3.45 (0.27)	3.42 (0.29)	3.78 (0.24)	3.54 (0.27)	3.95 (0.23)

**All means are adjusted for BDI-II, Brooding, and Reflection covariates; RNTs and FAEs are also adjusted for task accuracy. Recurring Negative Thoughts (RNTs) were rated on a scale of 1 (none at all) to 9 (an extreme amount), with 5 representing a moderate amount. Feelings After Errors (FAEs) were rated on a scale of 1 (extremely unpleasant) to 9 (extremely pleasant), with 5 representing neither pleasant nor unpleasant.**

#### Post-set Thoughts and Feelings

Table 2 also shows the mean ratings of RNTs and FAEs that participants reported experiencing during the general knowledge task.

##### Recurring negative thoughts

In general, all participants reported experiencing a fairly low frequency of RNTs (i.e., ratings of ∼3) across the general knowledge task. When submitting RNTs to our customized ANCOVA, we found a marginally significant two-way interaction involving Induction condition and Block, *F*(1, 47) = 2.88, *p* = 0.096, η*_p_*^2^ = 0.06, that subsumed a main effect of the Block factor, *F*(1, 47) = 16.56, *p* < 0.001, η*_p_*^2^ = 0.26. *Post hoc* testing of the two-way interaction revealed that in the Rumination condition only, participants indicated having *fewer* RNTs in Block 2 post-induction period than they did in the Block 1 pre-induction baseline (*p* = 0.001).

When considering RNT frequency in relation to pre-task individual differences measures, however, both the aforementioned two-way interaction and another two-way interaction involving Induction condition and trait Reflection, *F*(1, 47) = 6.43, *p* = 0.015, η*_p_*^2^ = 0.12, were qualified by a significant three-way interaction involving all three factors, *F*(1, 47) = 4.39, *p* = 0.042, η*_p_*^2^ = 0.09. *Post hoc* testing of the three-way interaction indicated a *positive* association between Reflection and RNTs in Block 1 only and for women the Rumination condition, β = 0.54, *t* = 2.93, *p* = 0.005, η*_p_*^2^ = 0.17. Trait Brooding, on the other hand, was found to only interact with Induction condition, *F*(1, 47) = 11.44, *p* = 0.001, η*_p_*^2^ = 0.09. Brooding predicted significantly more RNTs across the entire task for subjects, but only in the Distraction condition, β = 0.58, *t* = 2.93, *p* = 0.007, η*_p_*^2^ = 0.15. Moreover, Brooding unexpectedly predicted marginally *fewer* RNTs for those in the Rumination condition, β = -0.37, *t* = 1.80, *p* = 0.084, η*_p_*^2^ = 0.06.

##### Feelings after errors

Table 2 also shows that all participants generally reported feeling “somewhat unpleasant” (i.e., ratings of ∼4) after making an error during the general knowledge task. When submitting these subjective experience ratings to our customized ANCOVA, however, we found no effects of Induction condition. Rather, we found a significant main effect of Set, *F*(2, 94) = 6.08, *p* = 0.003, η*_p_*^2^ = 0.11, such that, compared to Set 1 ratings, all participants reported feeling more unpleasant after errors in Set 2 (*p* < 0.005) and Set 3 (*p* < 0.05), regardless of task block. There was also a marginal main effect of Block, *F*(1, 47) = 2.96, *p* = 0.092, η*_p_*^2^ = 0.06, which was led by participants reporting numerically more unpleasant feelings in Block 2 compared to Block 1, regardless of question set. With regard to the influence of trait and mood factors, we found only a significant main effect of trait Brooding, *F*(1, 47) = 5.59, *p* = 0.022, η*_p_*^2^ = 0.11, where those higher in this trait tendency had worse feelings about errors, regardless of condition or set. There was also a marginal main effect of BDI-II, *F*(1, 47) = 3.20, *p* = 0.080, η*_p_*^2^ = 0.06, but this will not be explored further.

##### Relationship between writing content and post-set thoughts and feelings

The rumination induction technique introduced in Block 2 was designed to activate brooding thoughts that would potentially carry over into participants’ subjective experience during the general knowledge task itself. To test for such a relationship, we examined the extent to which the proportion of RB content in the Rumination condition writing samples^5^ correlated with RNTs or FAEs, as a function of set. In support of the effectiveness of the induction, we observed a significant inverse relationship between proportion of RB content and FAEs during Set 2, *r*(27) = -0.575, *p* < 0.001, which is when negative FAEs peaked for all participants, regardless of condition. Thus, the degree to which participants were able to access and to express RB during the writing exercise was related to greater negative feelings about their mistakes, at least at a point in the task when those negative feelings were more likely to be salient for all participants. Interestingly, the proportion of self-reflective content (SR) in their writing was positively related to FAEs, *r*(27) = 0.606 = *p* < 0.001, consistent with a buffering effect of this more adaptive type of rumination on negative affect. These significant relationships between writing content and FAEs were maintained even when controlling for trait differences in Brooding and Reflection [correlation with RB: *r*(24) = -0.567, *p* = 0.002; correlation with SR: *r*(24) = 0.615, *p* < 0.001]. No significant relationships with FAEs were found during the other sets (all *p*s > 0.20) and no significant relationships were found for RNTs in any set (all *p*s > 0.13).

### Eye-Tracking Metrics

Table 3 shows the average duration of first fixation and total fixation to both types of competitive answer feedback (i.e., task-given correct answer and subject-given answer) during error trials.

**TABLE 3:** Average gaze fixation durations (in seconds) during error trials.

**Variable**	**Answer type**	**Condition**	**Pre-induction baseline**	**Post-induction period**
**FFD**				
	Correct answer			
		Rumination	0.310 (0.024)	0.301 (0.028)
		Distraction	0.265 (0.024)	0.356 (0.029)
	Incorrect answer			
		Rumination	0.185 (0.012)	0.207 (0.011)
		Distraction	0.201 (0.012)	0.199 (0.011)
**TFD**				
	Correct answer			
		Rumination	1.778 (0.073)	1.656 (0.082)
		Distraction	1.775 (0.075)	1.752 (0.084)
	Incorrect answer			
		Rumination	0.846 (0.051)	0.868 (0.052)
		Distraction	0.838 (0.053)	0.804 (0.053)

**FFD, First Fixation Duration; TFD, Total Fixation Duration.**

#### First Fixation Duration

An ANCOVA that included Induction condition, Block, and Answer type, along with the continuous RRS and mood variables, revealed a significant three-way interaction, involving all categorical factors, *F*(1, 43) = 12.31, *p* = 0.001, η*_p_*^2^ = 0.22. This higher-order interaction subsumed two marginally significant two-way interactions [Answer type by Block: *F*(1, 43) = 3.15, *p* = 0.083, η*_p_*^2^ = 0.07; Induction condition by Block: *F*(1, 43) = 3.50, *p* = 0.068, η*_p_*^2^ = 0.08], as well as significant main effects of Block, *F*(1, 43) = 6.68, *p* = 0.013, η*_p_*^2^ = 0.13, and Answer type, *F*(1, 43) = 49.26, *p* < 0.001, η*_p_*^2^ = 0.53. We also observed a marginally significant main effect of trait Brooding on FFDs in this analysis, *F*(1, 43) = 3.83, *p* = 0.056, η*_p_*^2^ = 0.08, that will not be explored further.

With regard to the main effect of Answer type, participants’ FFDs on the correct answer word were longer (*M* = 0.308, *SEM* = 0.017) than they were on reminders about their incorrect response (*M* = 0.198, *SEM* = 0.007), a relatively large difference that can be seen in the top half of Table 3. Due to this large overall difference, we will unpack the significant three-way interaction using a simple effects approach after first splitting by Answer type and conducting separate two-way mixed-measures ANCOVAs on FFDs to the subject-entered incorrect answer and FFDs to the task-provided correct answer.

When assessing FFDs on the incorrect response only, we found only a marginally significant two-way interaction between Induction condition and Block, *F*(1, 43) = 2.89, *p* = 0.096, η*_p_*^2^ = 0.06. Consistent with predictions, exploration of this interaction indicated that within the Rumination condition, FFDs on reminders of recent performance failures were longer during the post-induction period (i.e., Block 2) than they were during the pre-induction baseline (i.e., Block 1), although this effect did not survive *post hoc* Holm–Bonferroni corrections for multiple comparisons (uncorrected *p* < 0.05). In contrast, this same block-based comparison within the Distraction condition did not approach significance, nor did either of the condition-based comparisons within each block (all *p*s > 0.33).

**FIGURE 5:**
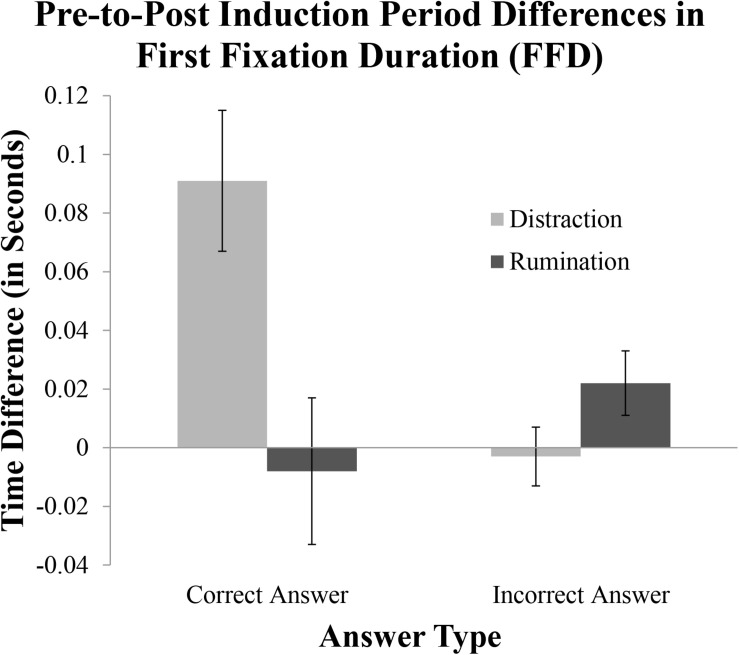
Pre-to-post induction differences in First Fixation Duration (FFD). Changes in FFD between the pre-induction baseline (i.e., Block 1) and post-induction period (i.e., Block 2) are plotted as difference scores (i.e., Block 2 FFD - Block 1 FFD). Difference scores are plotted as a function of Induction condition (Distraction in light gray; Rumination in dark gray) and Answer type (i.e., correct gray answer and incorrect red answer).

In contrast, when assessing FFDs on the correct answer, we found a significant Induction condition by Block interaction, *F*(1, 43) = 8.30, *p* = 0.006, η*_p_*^2^ = 0.16, which also subsumed a main effect of Block, *F*(1, 43) = 5.74, *p* = 0.021, η*_p_*^2^ = 0.12. Specifically, among those in the Distraction condition, FFDs on the correct answer were significantly longer during the post-induction period than they were during the pre-induction baseline (*p* < 0.005). This same block-based comparison within the Rumination condition was not significant, nor were any of the condition-based comparisons within each block (all *p*s > 0.18). Figure 5 illustrates the contrasting pre-post induction differences of the Rumination and Distraction conditions on both the correct answer compared to the reminder of the incorrect answer.

#### Total Fixation Duration

For this gaze metric, we found only a significant main effect of Answer type, *F*(1, 43) = 185.32, *p* < 0.001, η*_p_*^2^ = 0.81, indicating that all participants’ TFDs on the correct answer were much longer (*M* = 1.740, *SEM* = 0.053) than their TFDs on reminders about their incorrect response (*M* = 0.839, *SEM* = 0.035), and a significant main effect of Block, *F*(1, 43) = 6.05, *p* = 0.018, η*_p_*^2^ = 0.12, where TFDs observed in the post-induction period were *shorter* (*M* = 1.270, *SEM* = 0.033) than those seen in the pre-induction baseline (*M* = 1.309, *SEM* = 0.029). There were no overall differences or interactions involving the Induction condition factor (all *p*s > 0.15).

Interestingly, trait RRS predicted TFD, regardless of Induction condition. In particular, we observed a significant three-way interaction involving Answer Type, Block, and trait Brooding, *F*(1, 43) = 7.89, *p* = 0.007, η*_p_*^2^ = 0.16, which subsumed both a main effect of Brooding, *F*(1, 43) = 6.08, *p* = 0.018, η*_p_*^2^ = 0.12, and a two-way interaction involving Answer type and Brooding, *F*(1, 43) = 5.92, *p* = 0.019, η*_p_*^2^ = 0.12. Unpacking the three-way interaction, we found that within the pre-induction baseline only (i.e., Block 1), Brooding surprisingly predicted significantly longer TFDs on the *correct answer* feedback type, regardless of Induction condition, β = 0.50, *t* = 3.40, *p* = 0.001, η*_p_*^2^ = 0.20. No parameter estimates involving the relationship of Brooding with TFDs on reminders of participants’ *incorrect responses* in either Block 1 or Block 2 were significant (all *p*s > 0.27).

We also observed a significant three-way interaction involving Answer type, Block, and trait Reflection, *F*(1, 43) = 4.37, *p* = 0.43, η*_p_*^2^ = 0.09. *Post hoc* examination of parameter estimates again revealed an effect involving only the correct answer feedback type, and only within the pre-induction baseline (i.e., Block 1). However, this particular trait style of ruminative responsiveness surprisingly predicted significantly *shorter* TFDs to this novel corrective information for all subjects, β = −0.41, *t* = 2.89, *p* = 0.006, η*_p_*^2^ = 0.15. No parameter estimates involving the relationship of Reflection with TFDs on reminders of participants’ incorrect responses in either Block 1 or Block 2 were significant (all *p*s > 0.39). Finally, in contrast to the significant moderation of TFD effects by both trait RRS subtypes, pre-task BDI-II scores exhibited only a marginal interaction with Induction condition and Block, *F*(1, 43) = 2.87, *p* = 0.097, η*_p_*^2^ = 0.06, that will not be discussed further.

## Discussion

In the context of a challenging general knowledge retrieval task (see also Butterfield and Mangels, 2003; Whiteman and Mangels, 2016), the present study asked whether women induced into a state of rumination or distraction would allocate attention differently to reminders of their retrieval mistakes (i.e., incorrect answer) vs. new information (i.e., the correct answer), as measured by two metrics of gaze fixation duration – FFD and TFD. Both the rumination and distraction induction procedures involved completing handwritten narratives contextualized to be academically relevant. However, whereas the Distraction condition involved retrieval of the non-emotional details in the schedule of an average school day, the Rumination condition involved retrieval of details about an unresolved academic concern. On both our experimenter-quantified (i.e., writing content) and subjective self-report (i.e., post-writing experiential self-focus) measures, our findings support the conclusion that the women in this study carried out the writing exercises in induction-congruent ways, both replicating and extending previous studies (e.g., Roberts et al., 2013; Marin and Rotondo, 2017). Thus, we can be reasonably confident that our task-related findings can be interpreted from the assumption that participants had been successfully induced into states of either rumination or distraction.

Before discussing those task-related effects in detail, we first expand on the similarity between the pattern of RB and SR in our writing samples and those of Marin and Rotondo (2017), who examined ruminative content in 15 min writing samples taken once a day over 3 days. In Marin and Rotondo (2017), participants instructed to write about a recent, stressful experience maintained a relatively high degree of focus on the causes of that experience and related negative evaluations (i.e., RB) across the 3 days, with greater degrees of expression of this maladaptive form of rumination being associated with more negative self-focus and lower self-acceptance. However, by their third writing sample, participants also evidenced an increased degree of positive evaluation about their circumstances (i.e., SR). Both patterns are similar to that found in the present study and stand in contrast to studies where expressive writing has been found to be more beneficial for mood and behavior (e.g., Gortner et al., 2006; Sloan et al., 2008; Ramirez and Beilock, 2011). In particular, our prompts, which were aligned specifically with components of Martin and Tesser’s (1996) socio-cognitive theory of rumination, appeared to keep women brooding over their unresolved issues as they sequentially described their concern, the thoughts associated with it, and the time and energy they had spent unsuccessfully trying to resolve it. Only after the third time they had written about their unresolved situation were the women able to introduce more active reflection on how to move beyond it.

Despite the apparent effectiveness of the induction procedure, we did not find robust evidence for our hypothesis that being induced to ruminate versus distract increased attention to reminders about one’s errors. Although there was some evidence from planned comparisons that women in the Rumination condition increased their FFDs to the incorrect answer after the induction, this effect was not strong enough to survive correction for multiple comparisons. No other gaze duration effects specific to state Rumination were found. Thus, these findings provide weaker support than expected for both the impaired disengagement (Koster et al., 2011) and attentional scope theories of rumination (Whitmer and Gotlib, 2013). However, this may be related in part to the type of information presented. Past studies have primarily utilized passive viewing of negative faces or self-relevant words [e.g., passive viewing of emotional faces with no explicit task-based instruction (Duque and Vázquez, 2015; Owens and Gibb, 2017) and passive viewing of emotional or self-focused words in a simple target discrimination task (Grol et al., 2015; Southworth et al., 2017)].

Although we presented the incorrect answer in red, a color that some studies have shown to be implicitly arousing and negative (Elliot et al., 2007, 2009), the words themselves were not intrinsically negative. Instead, it was the internal construal of the meaning of this feedback in reference to achievement-related goals that might generate ruminative thoughts, something that may not be captured by gaze fixation. Gaze fixation metrics can only speak to where and how long the eyes dwell on the screen, but do not necessarily speak to what individuals are focusing on internally. Although models of eye movements and attention are often based on the principle that where one is looking is what one is thinking about (e.g., Just and Carpenter, 1976), at any given moment, the object(s) of internal and external focus can be different (Hunt and Kingstone, 2003), such as has been shown in the eye-tracking patterns of individuals who are mind wandering (Reichle et al., 2010).

Additionally, in our general knowledge task, participants had a choice between processing feedback information they already knew (i.e., that they had made a mistake) or updating their already existing knowledge by attending to the correct answer. The difference in information value between these two stimuli may have been sufficient to reduce the strength of any attentional biases toward their reminder of their error. Indeed, FFDs to reminders of recent performance failures were two-thirds the duration they spent fixating the novel corrective information, which is not surprising given the sensitivity of eye-tracking measures to word frequency and other lexical/semantic features (e.g., Reichle et al., 2003; Staub et al., 2010). Future studies where the competitive answer feedback represents the first time that participants learn of their response’s accuracy (i.e., eliminating the initial red or green circle indicating accuracy) may increase the sensitivity of our general knowledge task to such attentional-bias predictions by making sure the participant’s incorrect answer and the task-provided correct answer both provide new, although qualitatively different, information.

The effect of state rumination on FFDs to reminders of the participants’ errors may also have been stronger if we had been able to separately analyze FFDs associated with each set of questions in the post-induction period, rather than having to collapse across the three sets to achieve sufficient trial counts. Indeed, our measures of subjective experiences suggest that the rumination induction may have had a stronger influence on the first two sets compared to the third. Specifically, after the first writing exercise, we observed the largest differential between the Rumination and Distraction conditions on post-writing negative experiential self-focus. Although on that particular measure there was a regression toward neutral ratings for both Induction conditions after the second and third writing exercises, in the second set, a stronger relationship between the ruminative writing content and subjective task experiences emerged. Specifically, in this set, the participants in the Rumination condition who included more RB content in their writing sample also reported greater negative feelings about their task-related errors (FAEs). Interestingly, it was during this set of trials that FAEs appeared to be heightened for all participants, regardless of Induction condition (or block), suggesting that carryover from the writing exercise was greatest at a point in the task when the negative impact of repeated failure became the most salient. However, even though FAEs in the third and final set remained more negative overall, relative to the first set, the writing samples in the Rumination condition at this point in the post-induction period changed to include more of a putatively “adaptive” form of rumination—SR. Given evidence that SR in the writing exercise could reduce the sting of negative feedback (i.e., positive correlation with FAEs in Set 2), it is possible that by the third set, any selective attentional bias toward the reminder of the incorrect response in the Rumination condition, similarly, may have been mitigated by these adaptive influences.

Alternatively, the effects of rumination on feedback may simply be better explained by the inability to disengage from internally focused attention to the goal-based appraisal of the negative feedback rather than by overt attention to the external feedback itself. For example, in an event-related potential (ERP) study of trait rumination that used a similar general knowledge retrieval paradigm as in the current study (Whiteman and Mangels, 2016), we found that women higher in trait Brooding demonstrated evidence of more sustained attention to negative feedback, particularly as the task progressed and errors accumulated, as indexed by the magnitude and duration of a late positive potential (LPP) waveform over posterior scalp regions. Notably, the LPP is a putative ERP index of motivated attention to a visually evocative stimulus (Schupp et al., 2004; Foti and Hajcak, 2008) but can continue to be modulated by internal representations of emotional stimuli sustained even after stimulus offset (Hajcak and Olvet, 2008).

Additionally, one future way to evaluate this hypothesis using eye-tracking methodology would be to examine pupil dilation to the initial, centrally presented negative feedback (i.e., the red circle). Pupil dilation can provide an index of noradrenergically mediated arousal and internal processing effort (e.g., Eckstein et al., 2017), and previous studies have found increases in the extent and duration of pupil dilation to emotionally relevant information in relation to rumination (e.g., Siegle et al., 2003; Duque et al., 2014). For the present study, however, our interest was not in arousal, but in whether the induction of rumination would bias attention toward reminders of the mistake, rather than toward new information through which participants could correct their errors. For this question, we felt that gaze duration was a more appropriate measure. Additionally, when setting up our experiment, we found that the bright lighting conditions necessary to optimize gaze tracking made it difficult to optimally record pupillometry, which required dimmer lighting to allow for an appropriate range of pupillary responses. Given our primary interest was in selective attention to competitive feedback, we optimized our lighting for gaze tracking, precluding analysis of pupil activity.

Although our measures of gaze dwell time did not yield strong evidence for increased overt attention to reminders of errors in the Rumination condition, the Distraction condition reliably led to an increased FFD to the *correct* answer feedback type compared to the pre-induction baseline (i.e., Block 1). One interpretation of this finding is that writing about a neutral school day distracted the women away from any academic concerns existing either inside or outside of the task, leaving greater resources to attend to new information following errors. This increase in FFD on the correct answer could also reflect a general increase in intrinsic curiosity about this information, motivated by an interest in integrating this information into their existing knowledge base (cf. Kang et al., 2009). Thus, from a mechanistic perspective, distraction may therefore serve as an adaptive emotion regulation strategy (e.g., McRae et al., 2010), helping to down-regulate negative affect and redirect attention onto actions or objects of thought that are external to the self.

In comparison, the lack of similar findings in the Rumination condition could be another symptom of a less adaptive attentional focus. Indeed, some related work that used gaze fixation to index motivation toward attainment of a personally relevant goal found that individuals looked less at a goal-related stimulus if they believed the goal reflected by that stimulus was unattainable (Light and Isaacowitz, 2006). By this view, the instruction to ruminate about an unresolved academic concern may have primed women in our study to believe that an effort to resolve their poor performance was fruitless, and thus, any sustained overt attention to the correct answer would not be useful in this regard. Although a reliable decrease in FFD to the correct answer during the post-induction period would have given stronger evidence for this interpretation, we can at least conclude that the Rumination condition did not increase overt attention to the correct answer in the manner observed in the Distraction condition.

Finally, turning to our exploration of whether pre-task levels of trait Brooding and/or Reflection might influence subjective and objective measures within the task, we found a number of interesting findings. First and not unexpectedly, during the writing exercises, women who already had a trait tendency toward Brooding were more likely to describe their situation with phrases labeled as RB, regardless of whether they were writing about an unresolved academic issue or a neutral day. These findings are similar to those of Roberts et al. (2013), who also found that trait Brooding predicted significantly more reports of ruminative thoughts in their state Rumination condition. In contrast, a trait tendency to reflect seemed to buffer against the particular high levels of brooding content found in the Rumination condition, at least by the third time participants engaged in writing about their situation, which is when SR content increased for all women in the Rumination condition. Thus, when engaged in retrieval of autobiographical episodes, trait Brooding and Reflection tendencies appeared to either augment or buffer the expression of the more negative, moody brooding content in their writing samples, respectively.

Second, to the extent that trait RRS significantly affected gaze duration, it did so only for TFDs, and only in the pre-induction baseline, before any of the state effects described above unfolded. However, somewhat counterintuitively, Brooding predicted *increased* TFDs to corrective feedback, whereas Reflection predicted *decreased* TFDs to this information in that initial block. Unlike FFDs, however, which support initial lexical and semantic processing of the answers (Reichle et al., 2003; Staub et al., 2010), interpretation of TFDs in this task are less straightforward. There are multiple reasons why women might return to the correct answer component of the competitive feedback after fixations elsewhere. For example, the longer TFDs associated with greater trait Brooding might represent multiple short gaze fixations as they go back and forth from other areas of the screen, periodically reminding themselves of the correct answer, or a more sustained return to the correct answer as internal thoughts wander to task-relevant thoughts (i.e., “Why didn’t I put *that* answer?”) or even task-irrelevant thoughts (e.g., Reichle et al., 2010).

In contrast, consistent with the general association of trait Reflection with the desire to “take space” from one’s issues in order to proactively self-reflect (Treynor et al., 2003), the shorter TFDs associated with this particular RRS subcomponent could represent looking elsewhere on-screen (i.e., blank space, center of the screen in active preparation of the next question) once the correct answer had been initially processed. Unfortunately, the self-reports of RNTs do not provide much insight here, as they yielded state and trait rumination effects that were complex and difficult to interpret, possibly because RNTs had very low frequencies regardless of either condition or block. Whatever the reason for the opposing effects of trait Brooding and Reflection, however, the lack of interaction between trait RRS and state condition during the post-induction period suggests that, at least in this sample, effortful attempts to complete the written Rumination and Distraction narratives may have temporarily dominated the influence of trait tendencies on overt measures of attention in this task.

## Conclusion

Throughout college, many students will experience some type of impediment to attaining their academic goals, making them vulnerable to recurrent and self-focused thoughts as they try to minimize and resolve goal-state discrepancies. Theorists in both cognitive and clinical domains have identified a tendency to habitually ruminate in response to personal challenges and negative mood states as being integral to the development of depression, especially for women (e.g., Nolen-Hoeksema, 1987; Nolen-Hoeksema and Morrow, 1991; Watkins and Teasdale, 2001). Here, we demonstrated that even the simple process of being reminded of and writing about one of these unresolved academic situations was sufficient to at least temporarily increase otherwise mentally healthy women’s negative self-focus (e.g., greater endorsement of hopelessness), compared to writing about a neutral school day. Furthermore, although the ability of the induction to elicit RB writing content was related to trait rumination, the state induction itself appeared sufficient to override any influence of these trait tendencies on behavior, and least in this female sample that was not clinically depressed.

In our academically relevant general knowledge retrieval task, we found some evidence (from planned comparisons) for consequences of the Rumination condition in the form of increased initial dwell time (FFD) on reminders of their past mistakes, coupled with even stronger evidence for the benefits of the Distraction condition for increasing initial dwell time on potentially corrective information (see also Figure 5). Taken together, these findings provide not only some support for predictions from attentional theories of rumination predicting an exaggerated focus on negative, self-relevant information (Koster et al., 2011; Whitmer and Gotlib, 2013) but also the view that distraction can be a beneficial method of emotion regulation in the face of failure (cf. Nolen-Hoeksema et al., 2008; McRae et al., 2010). Even though both differences amounted to fractions of a second in gaze duration, these differences could still have implications for downstream learning, given that ERP studies using a variant of this general knowledge task have shown neural differences predicting successful encoding of correct answers starting as early as 300 ms after word onset (i.e., Butterfield and Mangels, 2003).

Despite intriguing results from this first-known study testing the influence of state rumination on the differential allocation of overt attention to feedback following failures, we have already described a number of ways in which future studies could improve study sensitivity. The functional relationship between differences in dwell time and error correction in this task is also important to establish. In at least one intentional encoding experiment, longer dwell time (as indexed by both FFD and TFD metrics) predicted greater subsequent recognition of verbal stimuli (Pazzaglia et al., 2014). Taken together with our current results, it would suggest that rumination could lead to better memory for one’s own mistakes whereas distraction would lead to better correction of those mistakes. We also acknowledge that our current findings are limited to a female sample, leaving open the question of how men would respond to our writing-based induction. Addressing these open issues would be valuable given the implications for developing interventions for students who, when facing significant academic difficulties, may experience states of rumination that interfere with optimal attention to learning resources needed to reach their academic goals.

## Data Availability Statement

The datasets generated for this study are available on request to the corresponding author.

## Ethics Statement

The studies involving human participants were reviewed and approved by the CUNY IRB. The patients/participants provided their written informed consent to participate in this study.

## Author Contributions

RW and JM conceived the research problem, method, and design, interpreted the data, and wrote the manuscript. RW ran the participants and analyzed the data. This research was conducted in partial fulfillment of the Ph.D. dissertation of RW. Both authors contributed to the article and approved the submitted version.

## Conflict of Interest

The authors declare that the research was conducted in the absence of any commercial or financial relationships that could be construed as a potential conflict of interest.

## Footnotes

1 Orthographic errors (i.e., misspelled but otherwise semantically accurate responses that overlapped orthographically with the correct answer to a degree of 75–99%) were excluded because they would likely lead to different patterns of eye movements (e.g., comparing letter for letter) than semantic errors. After excluding the participant outlier, the rate of orthographic errors in the remaining sample was very low (Rumination: *M* = 0.06, *SEM* = 0.006; Distraction: *M* = 0.06, *SEM* = 0.006), and this proportion did not differ as a function of Induction condition and/or Block (all *p*s > 0.11).

2 The preferred shortened form of the RRS was used, which has the Depression subscale removed (Treynor et al., 2003).

3 www.mangelslab.org/bknorms

4 We also considered whether it was necessary to include age or education as a covariate in our analyses, due to some marginal differences in these participant characteristics between Rumination and Distraction groups. We found that age correlated with task accuracy in the total sample (*r* = -0.29, *p* < 0.05), but because we had already partialed out variance due to differences in task accuracy in the eye-tracking and post-task surveys, we did not feel that it was necessary to further partial out variance due to age.

5 Floor effects on the levels of RB and SR in the Distraction condition precluded a corresponding analysis for that induction condition.
