# Supplementary material for: State and Trait Rumination Effects on Overt Attention to Reminders of Errors in a Challenging General Knowledge Retrieval Task
Source: Front Psychol. 2020 Sep 2;11:2094. doi: 10.3389/fpsyg.2020.02094 (PMC7492652; doi:10.3389/fpsyg.2020.02094)
Supplement: Supplementary file 1 [file Data_Sheet_1.DOCX]

1. *Condition-Specific Instructions*

**Rumination Condition:**

“Based on your responses so far, you encountered some difficulty answering the first block of questions. Although this difficulty is happening within the context of this research study, perhaps you have encountered difficulties in actual academic situations of your own life. During real-life academic difficulties, many students often report taking the time to think about the causes and meaning of their difficulties. Please take a moment to pause from the general knowledge questions to complete this second task that involves thinking about your academic difficulties in real life…

In this task, we want you to take some time to identify *a real, on-going, and unresolved academic concern* *in your own life* that has entered your mind once or twice, or maybe more, causing you to feel distressed across the past few weeks. If you cannot identify an academic concern from the past few weeks, it’s okay to identify one that started at some point during this semester, academic session, or school year. Still, it’s important that your concern is one that *remains unresolved and has entered your mind a lot, causing you distress across this particular period of time*.

Please hit the *Enter* key to continue…”

**Distraction Condition:**

“Based on your responses so far, you encountered some difficulty answering the first block of questions. Although this difficulty is happening within the context of this research study, perhaps you have encountered difficulties in actual academic situations of your own life. During real-life academic difficulties, many students often report taking the time to distract themselves from thinking about their difficulties. Please take a moment to complete this second task as a distraction from the general knowledge questions or any real- life academic difficulties you may be thinking about…

In this task, we want you to take some time to think about your academic schedule at Baruch College and identify a day across the past few weeks for which you can *remember with good accuracy what sorts of things you did while you were here on campus*. If no particular day stands out to you as memorable, just think back to a day for which you can remember what classes you attended, what friends you met up with between classes, what food you ate for lunch, and so on. Whatever day you choose, it’s important that the day be one for which *you can remember with good accuracy what sorts of events took place for you while you were at Baruch*.

Please hit the *Enter* key to continue…”

1. *Condition-Specific Examples*

**Rumination Condition:**

“Take a look at the following example of an unresolved academic concern that one participant gave permission for us to show you…”

*I’m enrolled in this course for my major right now where the material that’s been covered across the past few weeks has been really difficult for me to understand. I’ve repeatedly performed below my expectations on quizzes and assignments, and recently at night as I’ve tried to fall asleep I’ve found myself continuously wondering why this is happening to me, and why no one else seems to have the problems I’m having. What’s worse is that the exam grade I just got back was one of the lowest in the class, and since I spent more time studying than ever before, I’m now completely frustrated and pretty much can’t stop thinking about it.*

“Or consider this example of an unresolved concern that another participant gave permission for us to show you…”

*Across the past few weeks I’ve become preoccupied with what the students in this one class think of me. I’ve been part of a study group with some of them before, but recently I noticed that they formed a new study group without me. Across the past few class periods, I’ve caught myself mind- wandering about whether I know as much as they do. I decided a few days ago to ask if I could join them, but they basically just continued to give me the cold shoulder. Now since asking them, I’ve been feeling totally unappreciated and very concerned that my contributions don’t measure up to their standards. It’s been hard for me to keep the negative thoughts about it all from cycling over and over in my head.*

**Distraction Condition:**

“Take a look at the following example of a day at Baruch that one participant gave permission for us to show you…”

*Mondays are my longest day at Baruch since I have three classes that day. Psychology is first, and then later in the day I have my Accounting and Management classes, back to back. I’m choosing this past Monday to write about because in the morning I remember picking up some coffee with a friend at Starbucks before heading up to Psychology. Then in Psychology I remember we went over a huge exam that the professor handed back to us. Later I met a friend outside and we went to Bagel Express. In the afternoon, I had my Accounting class, and the professor let us out a little early. After my Management class later on I studied in the library for a few hours with three of my friends before getting on the subway to go home for the night.*

“Or consider this example of a day that another participant gave permission for us to show you…”

*Last Wednesday kind of stands out in my mind as being easy to remember. I got to Baruch earlier than usual that day since the subways were running smoothly. That gave me some extra time to finish my homework for my afternoon Math class. Psychology was my first class of the day though, and after that a bunch of us went out to get pizza at a place on 3^rd^ Ave that I had never been to before. After lunch I went to Math class, and then I always have some free time before Finance starts. But that day, I remember I got a text from my study group for my Psychology class, so I used my free time to meet up with them that day. After Finance, I went to the gym, and then went home.*

1. *Pre-Writing Rating Questions*

**Rumination Condition:**

1. Please rate how often you thought about the unresolved issue you have identified in your life ***while you were answering*** ***the most recent block*** of general knowledge questions.
2. Please rate how much the unresolved issue in your life concerned you while you were answering ***the most recent block*** of general knowledge questions.
3. Prior to thinking about your unresolved concern for the purpose of this research study today, please rate how often you’ve spent time thinking about your concern ***since it first started***.
4. Please rate how much your unresolved issue has been concerning to you ***at its worst***.

**Distraction Condition:**

1. Please rate how often you thought about the day you identified from your schedule ***while you were answering*** ***the most recent block*** of general knowledge questions.
2. Please rate how much the day you identified from your schedule concerned you while you were answering ***the most recent block*** of general knowledge questions.
3. Prior to thinking about the day from your schedule for the purpose of this research study today, please rate how often you’ve spent time thinking about that day ***since it happened***.
4. Please rate how much your schedule of events from that day has been concerning to you ***at its worst***.
5. *Induction-Related Writing Prompts*

**Rumination Condition:**

**PROMPT 1:** Now that you have identified your own unresolved academic concern, please take a few more moments to write your concern down on the lines below. Please refer to the tips below that will help guide you through this process. Please write about your concern as honestly and accurately as possible. When you are finished, please alert the experimenter. (See the attached pages again for student examples.)

Guidelines:

1. Use as much space as you need – your entry can be as long as the examples presented on the screen.
2. Avoid worrying about spelling or grammar; but please write legibly / clearly.
3. Avoid expressing too many personal feelings or emotions you may have about your concern.
4. Rather, as honestly and accurately as you can, provide a description of the concern itself.
5. You have plenty of time – the experimenter will check in with you in 3-5 minutes to see if you’re done.

**PROMPT 2:** In a few short sentences, given what you wrote about earlier, describe what sorts of RECURRING or REPEATED thoughts tend to pop into your head when your unresolved academic concern comes to mind. If you don’t usually experience any recurring or repeated thoughts, simply describe *the thoughts that* *most easily come to mind* about your unresolved concern.

For Example:

If you wrote earlier about currently doing poorly in a course for your major despite all your effort, you might say: “I can’t stop thinking about why this is happening to me.” Or “I often wonder ‘What’s the matter with me?’” And so on. Please write about all of the COMMON or RECURRING thoughts you tend to have as honestly and accurately as you can.

**PROMPT 3:** In a few short sentences, describe the AMOUNT OF TIME AND ENERGY you have invested in trying to resolve your on-going academic concern. Given that your concern is still unresolved, *what is your immediate reaction* to the amount of time and energy you have spent trying to resolve it?

For example:

If you wrote earlier about currently doing poorly in a course for your major despite all your effort, you might say: “I didn’t spend as much time studying as I needed to – and now that mistake has come back to haunt me.” Or “Even after all my effort, I still can’t see a light at the end of the tunnel – this is really frustrating.” And so on. Please write your actual thoughts about YOUR TIME AND EFFORT as honestly and accurately as you can.

**Distraction Condition:**

**PROMPT 1:** Now that you have identified a day from your own academic schedule at Baruch College, please take a few more moments to write down what you did while you were here on campus on the lines below. Please refer to the tips below that will help guide you through this process. Please write about your schedule as honestly and accurately as possible. When you are finished, please alert the experimenter. (See the attached pages again for student examples.)

Guidelines:

1. Use as much space as you need – your entry can be as long as the student examples provided here.
2. Avoid worrying about spelling or grammar; but please write legibly / clearly.
3. Avoid expressing personal feelings or emotions you may have about your schedule.
4. Rather, as honestly and accurately as you can, provide a description of the day you chose.
5. You have plenty of time – the experimenter will check in with you in 3-5 minutes to see if you’re done.

**PROMPT 2:** In a few short sentences, given what you wrote about earlier, describe the details of WHEN these events of your day occurred. That is, starting with the first event of the day that you mentioned and ending with the last, specify as precisely as possible *what time each event occurred and how long it lasted.*

For Example:

If you previously wrote about what classes you had and the lunch you ate, you might now say: “My first class started at 10:20 and ended early at 1:05. Then I ate lunch from 1:15 to 1:50.” And so on. Please write about WHEN THINGS HAPPENED as precisely as possible.

**PROMPT 3:** In a few short sentences, please write specific details about WHERE on campus (or around campus) the events you wrote about before took place. That is, starting with the first event of the day that you mentioned and ending with the last, specify as precisely as possible *the location where each event occurred*.

For Example:

If you wrote about what classes you had and the lunch you ate, you might say: “My first class was in the huge lecture hall on the 4th floor of the Vertical Campus. For lunch, we went to the cafeteria on the 1st floor and sat at one of the tables’ right outside the registers.” And so on. Please write about WHERE YOU WERE as precisely as possible.

1. *Writing Content Analysis*

To ensure accurate and consistent adherence to this coding system, the current author and a research assistant independently coded 25% of the 165 (3 writing samples x 55 participants) written narratives, similar what was done in the work of Marin & Rotondo (Marin & Rotondo, 2017). Cohen’s Kappa between the two independent coders was 0.740 (*p* < 0.05) for RB and 0.756 (*p* < 0.05) for SR. Any disagreements in codings were resolved through discussion, after which the current author then coded all remaining writing samples. During both reliability and later codings, each rater was blind to Induction condition and induction-related writing prompt.
